# Persistent Positivity of Reverse Transcriptase-Polymerase Chain Reaction Test among Patients with COVID-19 in Rural Teaching Hospital : A Descriptive Cross-sectional Study

**DOI:** 10.31729/jnma.7077

**Published:** 2021-11-30

**Authors:** Narayani Maharjan, Niresh Thapa, Bibek Pun Magar, Muna Maharjan, Jiancheng Tu

**Affiliations:** 1 Program & Department of Clinical Laboratory Medicine, Center for Gene Diagnosis, Zhongnan Hospital of Wuhan University, Wuhan, China and Department of Molecular Laboratory, Karnali Academy of Health Sciences, Jumla, Nepal; 2 Department of General Practice and Emergency Medicine, Karnali Academy of Health Sciences, Jumla, Nepal; 3 Department of Clinical Biochemistry, Karnali Academy of Health Sciences, Jumla, Nepal; 4 School of Nursing and Midwifery, Karnali Academy of Health Sciences, Jumla, Nepal; 5 Program & Department of Clinical Laboratory Medicine, Center for Gene Diagnosis, Zhongnan Hospital of Wuhan University, Wuhan, China

**Keywords:** *COVID-19*, *recurrence*, *reverse transcriptase-polymerase chain reaction*, *SARS-CoV-2*, *viral shedding*

## Abstract

**Introduction:**

The persistence positivity detected for severe acute respiratory syndrome coronavirus 2 ribonucleic acid by real-time reverse transcriptase-polymerase chain reaction test in asymptomatic coronavirus disease 2019 positive patients has attracted a lot of attention. There is limited data on the duration of viral shedding. We aimed to determine the proportion of coronavirus disease patients with persistent positivity of real-time reverse transcriptase-polymerase chain reaction test in a teaching hospital of Nepal.

**Methods:**

A descriptive cross-sectional study was conducted using medical records from May to September 2020 in a teaching hospital of Nepal. The study was approved by the Institutional Review Committee of Karnali Academy of Health Sciences (Reference no 077/078/03). Convenient sampling method was used. Data was analysed by Statistical Package for the Social Sciences. Point estimate at 90% Confidence Interval was calculated along with frequency and proportion for binary data.

**Results:**

Of the total 95 cases, 9 (9.5%) (4.6-14.4 at 90% Confidence Interval) cases were repeat positive after achieving the first negative. The mean day required of achieving the last negative for the repeat positive group was 62.11±3.95, range (60-70 days). The mean time duration for the virus shedding was found to be 20.43±12.19 days (range 7-60 days) after the first positive test result.

**Conclusions:**

This study concludes that there might be a persistent positivity of the polymerase chain reaction test among patients with COVID-19. The majority of the patients were test positive for 8-14 days, and some were positive till 60-70 days.

## INTRODUCTION

The outbreak of COVID-19 caused by severe acute respiratory syndrome coronavirus 2 (SARS-CoV-2) has become a global public health emergency, including Nepal.^1^ With the rise in cases, there are several reports on persistent or recurrent positivity of reverse transcription-polymerase chain reaction (RT-PCR) test among patients with COVID-19.^2-9^ This persistence of SARS-CoV-2 RNA virus in body fluids of patients with COVID-19 may increase the potential risk of viral transmission.^10^

The duration of persistence of SARS-CoV-2 virus has significant clinical and epidemiological implications.^3^ However, the viral detection period in mild or asymptomatic patients with COVID-19 is still unclear. Hence, to reduce the risk of viral transmission, it is essential to know the duration of viral shedding to determine a re-testing and de-isolation plan of COVID-19 patients.^11^

Thus, we aimed to determine the proportion of COVID-19 patients with persistent positivity of the RT-PCR test in a rural teaching hospital, Nepal.

## METHODS

A descriptive cross-sectional study was conducted in Karnali Academy of Health Sciences (KAHS)-Teaching Hospital, Jumla, Nepal from May to September 2020. Total of 95 participants were enrolled in the study using convenience sampling. Patients with COVID-19 who were in isolation for more than 7 days in the hospital were recruited for this study. This study was approved by the Institutional Review Committee (IRC) of KAHS (IRC approval reference number: 077/078/03) Jumla, Nepal. Patients who were in an isolation ward and positive for RT-PCR test for more than one time were included in the study. The participants who refused to stay in isolation for more than 7 days or incomplete data were excluded. Convenience sampling was done and the sample size was calculated as,

n = Z^2^ × p × q / e^2^

  = (1.645)^2^ × 0.5 × (1-0.5) / 0.09^2^

  = 84

where,

n= required sample size,
Z= 1.645 at 90% Confidence Interval (CI),
p= 50%, taken for maximum sample size
q= 1-p
e= margin of error, 9%

Therefore, the minimum sample size calculated was 84. Non-response rate was adjusted at 10% of the sample size, the calculated sample size was 93. However, a total of 95 samples were enrolled in this study.

The data was collected from the medical record book and the RT-PCR report was collected from the PCR laboratory of KAHS-Teaching Hospital. A standard format was prepared to collect the information such as date of hospital admission, hospital number, address, age, gender, ethnicity, RT-PCR test report, and duration of isolation. The nasopharyngeal and oropharyngeal specimens had been collected from the participants for detection of SARS-CoV-2 virus. The RT-PCR test was performed as per interim guidance and protocol of WHO and National Public Health Laboratories (NPHL), Nepal for SARS-CoV-2 detection in the hospital PCR laboratory.^12,13^

The data were collected and sorted using Microsoft Excel (Microsoft Corporation, New York, USA) software; Statistical Package for the Social Sciences (SPSS version 16.0; IBM Analytics, New York, USA) software was used for the statistical analysis. Descriptive statistics were reported as frequency, mean, and percentage.

## RESULTS

Total of 95 patients with a positive SARS-CoV-2 admitted to the isolation ward of KAHS-Teaching Hospital were recruited in this study. In this study, 9 out of 95 (9.5%) 4.6-14.4 at 90% CI) cases were repeat positive for the RT-PCR test after achieving the first negative. All of them were male. In this group, 7 out of 9 (78%) were again positive after 47^th^/48^th^ day from the first positive. Similarly, 2 out of 9 (11% each) in 49^th^ and 60^th^ day respectively. The mean day of repeat positive for this group was 49.33±4.03, range (47-60 days). In this group of repeat positive cases, 6 out of 9 (67%) of patients achieved the last negative result on the 60th day from the first positive and the remaining 3 cases achieved the last negative result on 61^st^, 68^th^, and 70^th^ day. The mean day required of achieving the last negative for the group was 62.11±3.95, range (6070 days) (Table 1).

**Table 1:** Duration of recurrent RT-PCR positive cases (n= 9).

Repeat Positive Days	n (%)	Last Negative Day	n (%)
47	2 (22)	60	6 (67)
48	5 (56)	61	1 (11)
49	1 (11)	68	1 (11)
60	1 (11)	70	1 (11)

Among 95 positive cases, 37 (38.9%) and 33 (34.7%) patients were within the age group of 30-40 and 21-30 respectively. Under the age of 10, there were only 3 (3.2%) patients and over the age of 60, only 2 (2.1%). The mean age of patients was found to be 31.85±11.3, and 77 (81.1%) were male. Most of them 41 (43.2%) belonged to the Chhetri ethnic group (Table 2).

**Table 2:** Characteristics of patients (n=95).

Characteristics	n (%)
**Age (Years)**	
<10	3 (3.2)
11-20	7 (7.4)
21-30	33 (34.7)
30-40	37 (38.9)
41-50	7 (7.4)
51-60	6 (6.3)
>60	2 (2.1)
Mean age±SD	31.85±11.3
**Gender**	
Male	77 (81.1)
Female	18 (18.9)
**Ethnicity**	
Brahmin	15 (15.8)
Chhetri	41 (43.2)
Janajati	12 (12.6)
Thakuri	10 (10.5)
Dalit	12 (12.6)
Others	5 (5.3)

The mean time duration for SARS-CoV-2 RNA shedding was found to be 20.43±12.19 days (range 7-60 days) after the first positive test result. Only 34 (35.8%) of the patients achieved a negative test within 8-14 days after the first positive test and about 19 (20%) of patients required 29 and above days to achieve a negative test after the first positive test result (Table 3).

**Table 3:** Duration of RT-PCR positivity of patients (n=95).

Duration (Days)	n (%)
≤7	3 (3.2)
8-14	34 (35.8)
15-21	29 (30.5)
22-28	10 (10.5)
29 and above	19 (20.0)
Mean Duration	20.43±12.19
Range	7-60 days

About 26 (27%) and 24 (25%) of the patients within the age group of 20-45 achieved negative results in 8-14 days and 15-21 days respectively. Only 18 (18.9%) of patients required a longer duration of 29 days and above to achieve a negative test result. In male, the highest of 24 (25.3%) patients required 15-21 days to achieve a negative test result. In female, 12 (12.6%) of patients achieved negative result within 8-14 days (Table 4).

**Table 4:** Characteristics of patients in relation with positivity duration of SARS-CoV-2.

Characteristics	< 7 days	8-14 days	15-21 days	22-28 days	> 29 days
Age					
< 19	1 (1.1)	5 (5.3)	0	0	0
20-45	2 (2.1)	26 (27.4)	24 (25.3)	10 (10.5)	18 (18.9)
46-60	0	2 (2.1)	4 (4.2)	0	1 (1.1)
> 61	0	1 (1.1)	1 (1.1)	0	0
Gender					
Male	3 (3.2)	22 (23.2)	24 (25.3)	10 (10.5)	18 (18.9)
Female	0	12 (12.6)	5 (5.3)	0	1 (1.1)
Ethnicity					
Brahmin	0	6 (6.3)	6 (6.3)	0	3 (3.2)
Chhetri	3 (3.2)	15 (15.8)	13 (13.7)	3 (3.2)	7 (7.4)
Janajati	0	5 (5.3)	1 (1.1)	2 (2.1)	4 (4.2)
Thakuri	0	3 (3.2)	6 (6.3)	0	1 (1.1)
Dalit	0	4 (4.2)	3 (3.2)	2 (2.1)	3 (3.2)
Others	0	1 (1.1)	0	3 (3.2)	1 (1.1)

## DISCUSSION

Patients with COVID-19 who showed persistently positive RT-PCR test for SARS-CoV-2 have attracted a lot of attention. Some previous studies have evaluated follow-up RT-PCR results for SARS-CoV-2 among the patients who were recovered from COVID-19.^9,14,15^

We determined the proportion of COVID-19 patients with persistent positivity of the RT-PCR test in our study. In this study, we estimated the time duration for positive patients with COVID-19 to clear SARS-CoV-2 RNA virus shedding.

In the group of repeat positive cases, our study has shown that 9.5% (n=9) had repeat positive RT-PCR results for SARS-CoV-2 after the first negative result with a mean duration of repeat positive was 49 days (range 47-60 days). Several studies reported re-detectable positive cases after discharge.^9,14^ One retrospective study in Wuhan, China also reported 7.4% (8/108) were repeat positive.^5^ Further, a repeat positive case was reported in a 62 years old Japanese man. He had twice positive and negative test results over 48 days of hospitalization.^16^ These findings confirmed that a certain proportion of patients may still experience conversion and prolonged RT-PCR positivity regardless of any symptoms.

The reason for the recurrence of SARS-CoV-2 RNA positivity remains controversial. RT-PCR has been widely employed in diagnosing viral infections and has yielded few false-positive results.^17^ We hypothesized that the reason for repeat RT-PCR positive cases might be due to the detection of viral gene fragments without active viral replication or residual "dead" viruses. Also, based on previous studies, positivity may be associated with underlying diseases, clinical status, host immune function, sampling, processing and detection.^5,9,18,19^ Furthermore, the observed false-negative results have been associated with the quality of the kit as the lower limit of detection (LOD) of commercial RT-PCR kits is relatively high.^14^

In the study, we found a mean duration of 20 days with a range of 7-60 days to achieve a negative RT-PCR result. Hence, the results evidenced that most patients tested positive for more than 2 weeks to achieve the first negative results in an agreement with Carmo A. et al.^11^ About 35% of patients achieved negative results in 8-14 days and about 30% required 15-21 days. These results may suggest that in a situation of lack of testing resources, the patient might be re-tested only after the 20th day from the first positive test. As many other investigations also suggest for this.^6,11,20-23^

In the present study, the majority of enrolled cases were asymptomatic except 7 cases who had mild symptoms. There was not any mortality case found during this study period. Only 2 cases required oxygen, one pregnant lady with heart disease and another with age over 60 years. And also, there was no requirement of using a ventilator in this group of the study population. Our findings suggest that asymptomatic COVID-19 positive individuals can remain SARS-CoV-2 RT-PCR positive for a prolonged period of time. As the COVID-19 positive cases were asymptomatic, further laboratory tests or radiological investigation was not done.

Male patients were positive for a longer duration. It is difficult to compare and explain. Nevertheless, this could be due to the larger number of male patients and possibly other characteristics such as smoking, comorbid conditions which was out of the scope of this study.

The study had some limitations. This is a singlecentered study with a small size of positive COVID-19 patients. We were unable to find out the risk factors associated with positive patients as most of the cases were asymptomatic. Also, we were not able to perform viral culture which is the gold standard for viral detection with infectivity.

## CONCLUSIONS

This study concludes that there might be a prolonged period of persistence of SARS-CoV-2 RNA among patients with COVID-19. The majority of the patients were RT-PCR test positive for 8-14 days, and some were positive till 60-70 days among repeat positive cases.
